# Failure to Suppress Progression in Clear Cell Renal Cell Carcinoma Associated with NCOA7 Low Expression Revealed Through Radiogenomic Analysis

**DOI:** 10.3390/genes16040386

**Published:** 2025-03-28

**Authors:** Federico Greco, Marco Cataldo, Bruno Beomonte Zobel, Carlo Augusto Mallio

**Affiliations:** 1 Department of Radiology, Cittadella della Salute, Azienda Sanitaria Locale di Lecce, Piazza Filippo Bottazzi, 2, 73100 Lecce, Italy; 2 Research Unit of Radiology, Department of Medicine and Surgery, Università Campus Bio-Medico di Roma, Via Alvaro del Portillo, 21, 00128 Roma, Italy; b.zobel@policlinicocampus.it (B.B.Z.); c.mallio@policlinicocampus.it (C.A.M.); 3 Apphia s.r.l., Via per Monteroni, 73100 Lecce, Italy; marco.cataldo@apphia.it; 4 Fondazione Policlinico Universitario Campus Bio-Medico, Via Alvaro del Portillo, 200, 00128 Roma, Italy

**Keywords:** aggressiveness, clear cell renal cell carcinoma, computed tomography, NCOA7, radiogenomics, tumor progression

## Abstract

**Background/Objectives**: Radiogenomics investigates the relationship between imaging features and genomic characteristics, offering a non-invasive approach to studying tumor biology. Nuclear receptor coactivator 7 (*NCOA7*) is a conserved nuclear receptor coactivator with potential prognostic relevance in clear cell renal cell carcinoma (ccRCC). This study aims to evaluate the radiogenomic features associated with *NCOA7* low expression in ccRCC patients and its correlation with tumor aggressiveness. **Methods**: A cohort of 209 ccRCC patients was analyzed using genomic data from The Cancer Genome Atlas and imaging features from The Cancer Imaging Archive. Imaging characteristics were assessed through computed tomography scans, focusing on tumor size, margins, necrosis, growth patterns, and other radiological features. Statistical analyses, including Student’s *t*-test and Pearson’s Chi-square test, were used to examine associations between *NCOA7* expression and clinicopathological or radiological features, with significance set at *p* < 0.05. **Results**: *NCOA7* low expression was identified in 66.03% of patients and significantly associated with older age (*p* = 0.001), higher tumor grade (*p* = 0.015), advanced American Joint Committee of Cancer stage (*p* = 0.006), collateral vascular supply (*p* = 0.014), ill-defined margins (*p* = 0.016), tumor necrosis (*p* = 0.002), exophytic growth pattern ≥50% (*p* = 0.002), and perinephric fat stranding (*p* = 0.027). **Conclusions**: These findings indicate a radiologic phenotype suggestive of increased tumor aggressiveness. *NCOA7* low expression correlates with aggressive radiologic and clinicopathological features, underscoring its potential as a biomarker for poor prognosis in ccRCC. Radiogenomic integration provides insights into tumor behavior and aids in developing therapeutic strategies.

## 1. Introduction

Radiogenomics refers to the correlation between imaging features and genomic characteristics [1,2]. Imaging provides a macroscopic representation of molecular processes, collectively termed radiologic phenotypes [1,2]. The Cancer Genome Atlas (TCGA) Research Network includes a wealth of data on gene expressions and mutations associated with clear cell renal cell carcinoma (ccRCC) [3,4].

Radiogenomic analysis offers several significant advantages. Unlike biopsy sampling, it is a non-invasive technique and avoids the risks and complications associated with biopsies. While biopsy samples provide information only about the specific tissue extracted, radiogenomic analysis evaluates the entire tumor volume, enabling the capture of genomic data for the whole neoplasm. This comprehensive approach is crucial for understanding tumor heterogeneity, which is key to accurate prognosis. Biopsy samples may miss critical gene expressions or mutations essential for designing targeted therapies for effective disease treatment [5,6,7,8,9,10,11].

Radiogenomics also enables monitoring of the “genomic landscape” over time during patient follow-up, assessing how genomic variations respond to therapies. Moreover, it allows the simultaneous analysis of multiple lesions. Imaging techniques can quantify body composition and link it with genomic data [5].

Nuclear receptor coactivator 7 (*NCOA7*), also referred to as estrogen receptor (ER)-associated protein of 141 kDa, is a conserved nuclear receptor coactivator expressed in a tissue-specific manner. It has been demonstrated to interact with ERα, enhancing the transcriptional activity of the receptors it associates with [12]. ERα plays a significant role in abnormal cell proliferation, inflammation, and the development of malignant tumors, as well as impacting the tumor’s response to targeted therapeutic interventions [13]. Tests assessing the effects of *NCOA7* overexpression were performed in cellular and mouse models to elucidate its role and mechanism in suppressing the progression of ccRCC [14]. *NCOA7* expression was found to be reduced across all three subtypes of renal cell carcinoma, but it held significant prognostic relevance specifically for ccRCC patients [14]. Overexpression of *NCOA7* suppressed the proliferation, invasion, and metastasis of ccRCC cells both in vitro and in vivo [14]. On a mechanistic level, *NCOA7* inhibited the mitogen-activated protein kinase/extracellular signal-regulated kinase signaling pathway, regulating epithelial–mesenchymal transition and apoptosis, thereby curbing ccRCC progression [14]. By suppressing tumor growth and metastasis through the mitogen-activated protein kinase/extracellular signal-regulated kinase pathway, *NCOA7* emerges as a promising prognostic marker and potential therapeutic target for ccRCC [14].

To date, the radiogenomics of *NCOA7* expression in ccRCC patients has not been evaluated. This retrospective study aims to investigate the computed tomography (CT) findings associated with *NCOA7* expression in ccRCC patients. Based on its activity, it is hypothesized that low *NCOA7* expression may be associated with an aggressive radiologic phenotypic pattern. We propose that a CT-based approach could provide valuable insights into *NCOA7* expression in ccRCC.

## 2. Materials and Methods

### 2.1. The Cancer Genome Atlas

TCGA, supported by the National Cancer Institute and the National Human Genome Research Institute, serves as a comprehensive repository of genetic alterations across more than 20 cancer types, including ccRCC. Tissue samples were provided by participating institutions, each obtaining institutional review board approval beforehand. These samples underwent extensive multiplatform genomic analysis and characterization. Additionally, The Cancer Imaging Archive, funded by the National Cancer Institute, is an anonymized repository of medical imaging data in DICOM format. Pretreatment imaging data from The Cancer Imaging Archive are linked to tissue samples from TCGA using a unique identifier, making both datasets publicly available for download.

In TCGA, gene expression analysis follows a well-established pipeline to ensure consistency and accuracy. Typically, gene expression data is generated using RNA sequencing (RNA-Seq) technology, often on platforms like Illumina. The raw sequencing data is collected in the form of FASTQ files, which are then preprocessed to remove low-quality reads and adapters. The next step involves aligning these reads to the human reference genome, commonly GRCh38, using tools like STAR or HISAT2. This alignment ensures that the sequences are accurately mapped to their corresponding genomic locations.

Once the reads are aligned, the gene expression levels are quantified. This is done by counting the number of reads mapped to each gene, using specialized tools such as HTSeq or FeatureCounts. The expression levels are then reported in various metrics, including raw read counts, fragments per kilobase of transcript per million (FPKM) mapped reads, transcripts per million (TPM), or through methods like RNA-Seq by expectation maximization, which estimate transcript abundance.

To make gene expression data comparable across samples, normalization techniques are applied. One of the most common methods is calculating FPKM or TPM, which account for differences in sequencing depth and gene length. Other approaches include upper quartile normalization or median of ratios, which help adjust for variations that could otherwise introduce biases.

An essential step in TCGA’s gene expression analysis is thresholding, which distinguishes between high and low expression levels. Typically, thresholds such as FPKM greater than 1 or TPM greater than 1 are used to classify genes as “expressed” rather than “non-expressed”. In some cases, z-score normalization is applied to classify gene expression as “high” or “low” based on how far they deviate from the average expression level. Alternatively, percentile-based thresholding, such as using the upper quartile, may be employed to define high expression.

To minimize technical variability, TCGA applies batch effect correction methods, such as ComBat from the sva package, to harmonize data from different batches or experimental conditions. Finally, the processed and normalized gene expression data are made publicly available through platforms like cBioPortal and the Genomic Data Commons, allowing researchers to explore and analyze the data further [15].

### 2.2. Imaging Features

The imaging characteristics assessed through a CT-based approach for each case of ccRCC include: tumor size (measured in mm), composition (classified as solid or cystic), margin (well-defined or ill-defined), tumor necrosis (evaluated only for solid tumors as 0%, 1–33%, 34–66%, or >66%), growth pattern (categorized as endophytic, <50% exophytic, or ≥50% exophytic), presence of calcifications (absent or present), tumor laterality (left or right kidney), evidence of collateral vascular supply (e.g., macroscopically visible enlarged renal capsular veins in CT or magnetic resonance imaging studies), intralesional hemorrhage, infiltration, collecting system invasion, hydronephrosis, renal artery thrombosis, and renal vein thrombosis [5,16,17,18].

Additional CT findings included Gerota’s fascia thickening (noted as present or absent), and the presence or absence of perirenal fat stranding [5,17,18].

Tumor dimensions were determined by measuring the largest axial diameter in post-contrast images [5,16,17,18]. Margins were considered well-defined when more than 90% of the tumor’s circumference exhibited a sharp, “pencil-thin” appearance in postcontrast scans, including its interface with renal parenchyma, the collecting system, or surrounding fat tissue [5,16,17,18]. This evaluation was conducted using a window with settings of width 400 and level 50 [5,16,17,18]. Tumors were categorized as cystic if at least 50% of their volume was composed of well-delineated cystic spaces displaying fluid attenuation (≤20 HU). Tumors with a cystic component comprising less than 50% of the total volume or completely absent were classified as solid [5,16,17,18].

Necrosis, analyzed during nephrographic or excretory phases, was identified as hypodense regions without contrast enhancement and without clearly demarcated walls [5,16,17,18]. This characteristic distinguishes necrosis from cystic areas [5,16,17,18]. Calcifications were identified as high-density areas or plaques, with ambiguous cases evaluated based on maximum HU values exceeding 60 HU [5,16,17,18]. Intralesional hemorrhage was identified by the presence of regions with blood density HU values (+30 to +80) [5,16,17,18]. In cases where hemorrhagic regions and calcifications exhibited overlapping HU values, morphology was evaluated by two radiologists specializing in oncological imaging (F.G., 9 years of experience; C.A.M., 13 years of experience) to distinguish the two [5,16,17,18].

Tumor infiltration, observed in postcontrast phases, was defined by the extension of tumor tissue into adjacent healthy tissues [5,16,17,18]. Hydronephrosis, determined from postcontrast images, was identified as dilation of the urinary tract [5,16,17,18]. Renal artery or vein thrombosis was diagnosed from postcontrast scans as an intraluminal defect indicative of thrombus formation [5,16,17,18]. Collecting system invasion, evaluated in the excretory phase of postcontrast scans, was identified as the presence of tumor tissue causing filling defects within the collecting system [5,16,17,18]. Imaging features were analyzed using the Horos v.4.0.0 RC2 software. All the features were evaluated by the consensus of two radiologists (F.G. and C.A.M., with 9 years and 13 years of experience, respectively), who were blinded to the genomic and clinical–pathological data [5,16,17,18].

### 2.3. Statistical Methods

Descriptive statistics included frequencies and proportions for categorical variables. The relationship between *NCOA7* expression (low vs. high) and clinicopathological characteristics, as well as CT-based tumor features was investigated. Student’s *t*-test and Pearson’s Chi-square test were used to examine the statistical significance of differences in medians and proportions among the patient cohort stratified according to *NCOA7* expression. All tests were two-sided with a level of significance set at *p* < 0.05.

The decision to use Student’s *t*-test in this study was based on the need to compare means between two independent groups (low vs. high *NCOA7* expression) within a large sample size of 209 patients. The *t*-test is particularly appropriate when analyzing differences between group means, provided that the data distribution is approximately normal and the variability within groups is consistent. This approach allows for a robust evaluation of statistical significance, making it a suitable choice for our analysis. To ensure the validity of the *t*-test, we carefully assessed the independence of samples, the normal distribution of data, the homogeneity of variances, and the use of a metric scale for quantitative variables.

## 3. Results

In a cohort of 209 patients, low *NCOA7* expression was identified in 66.03% of cases, whereas 33.97% displayed high expression (Table 1). Significant differences in clinicopathological and CT-based tumor features were observed based on *NCOA7* expression. Patients with low *NCOA7* expression were older, with a median age of 61 years compared to 56 years in the high group (*p* = 0.001). Tumor grade and stage also varied significantly; low-grade tumors (G1-2) were more frequent in the *NCOA7*-high expression group (52.11%) compared to the low expression group (34.06%), while high-grade tumors (G3-4) were more prevalent in the low expression group (65.94%) (*p* = 0.015). Similarly, early-stage tumors (Stage I) were more common in *NCOA7*-high expression cases (64.29%) compared to low expression ones (44.20%), while advanced tumors (Stage IV) were predominantly observed in the low group (20.29% vs. 4.29%, *p* = 0.006).

CT imaging features also highlighted distinctions. Collateral vascular supply was more frequently observed in the low expression group (63.36%) compared to the high expression group (43.94%, *p* = 0.014). Tumor margins were more likely to be well-defined in *NCOA7*-high expression cases (76.47%) compared to the low group (58.21%, *p* = 0.016). Additionally, tumor composition showed a significant association, with solid tumors being more prevalent in the low group (97.01%) versus the high expression group (83.82%), while cystic tumors were more common in the high expression group (14.71%) (*p* = 0.004).

Further, tumor necrosis was significantly different between groups, with higher rates of necrosis >66% in low expression cases (13.43%) compared to high expression ones (4.41%, *p* = 0.002). Growth patterns also varied; exophytic growth patterns (≥50%) were more prevalent in low expression cases (72.39%), while high expression was associated with less exophytic growth (47.14%, *p* = 0.002). Finally, perinephric fat stranding was significantly associated with low expression (39.5%) compared to high expression cases (14%, *p* = 0.027).

**Figure 1:**
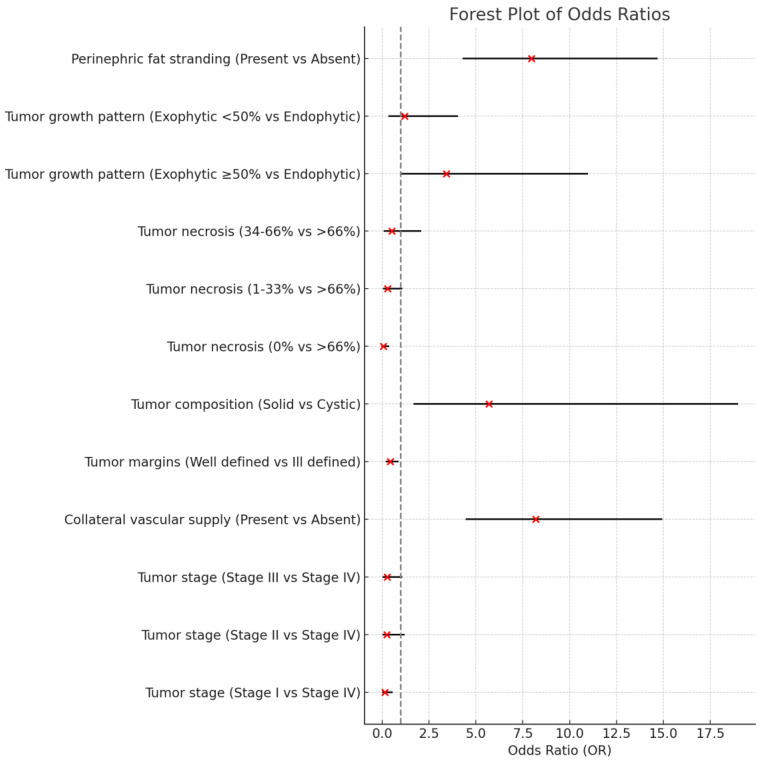
This forest plot visualizes the odds ratios (OR) for various clinical–pathological and CT-based features associated with NCOA7 expression. The plot includes comparisons between different levels of each variable. The red dots represent the odds ratios, while the horizontal lines indicate the 95% confidence intervals (CI). The vertical dashed line at OR = 1 represents the point of no effect, meaning values crossing this line indicate no significant association. The variables are organized as follows: tumor stage (Stage I, II, III, IV)—comparison between each stage and Stage IV. Collateral vascular supply—presence versus absence. Tumor margins—well-defined versus ill-defined. Tumor composition—solid versus cystic. Tumor necrosis (0%, 1–33%, 34–66%, >66%)—comparison between each necrosis level and the highest level (>66%). Tumor growth pattern (endophytic, exophytic <50%, exophytic ≥ 50%)—comparison between exophytic patterns and endophytic pattern. Perinephric fat stranding—presence versus absence. Significant associations are those whose confidence intervals do not cross the vertical line at OR = 1. Odds ratios greater than 1 indicate a higher likelihood of the outcome in the first group compared to the reference, while ratios less than 1 indicate a lower likelihood.

These findings underscore that tumors with *NCOA7* low expression are characterized by associations with older age, higher tumor grade, American Joint Committee of Cancer (AJCC) tumor stage, and more aggressive radiological features, highlighting the potential of *NCOA7* low expression as a marker for more aggressive tumor behavior and poorer prognosis in ccRCC. The results of the radiogenomic features are represented in Figure 1.

## 4. Discussion

**Figure 2:**
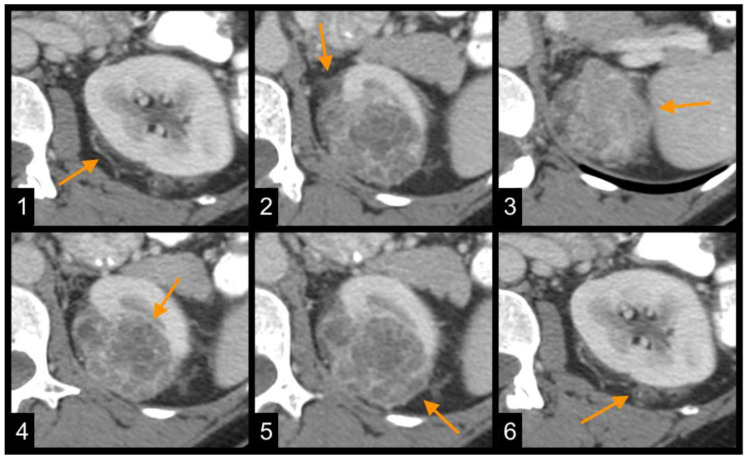
Representative CT images illustrating radiogenomic features of *NCOA7* low expression in ccRCC: (1) collateral vascular supply (arrow); (2) ill-defined tumor margins (arrow); (3) solid tumor composition (arrow); (4) tumor necrosis (arrow); (5) exophytic growth pattern ≥50% (arrow); (6) perinephric fat stranding (arrow).

In this work, we evaluated radiological features of *NCOA7* low expression in ccRCC patients. A significant association was found with age, tumor grade, more advanced AJCC tumor stage, collateral vascular supply, ill-defined margins, tumor composition, tumor necrosis, exophytic growth pattern ≥50%, and perinephric fat stranding (Figure 1). These findings suggest that *NCOA7* low expression correlates with radiologic phenotype indicative of increased tumor aggressiveness (Figure 2).

*NCOA7* expression was observed to be decreased in ccRCC tissues. Bioinformatic evaluations revealed the prognostic relevance of *NCOA7* specifically for ccRCC [14]. The levels of *NCOA7* expression were strongly associated with the clinicopathological features of patients, showing reduced expression in cases with high-grade and advanced-stage ccRCC [14]. This study identified an inverse relationship between *NCOA7* expression and the tumor N and M stages, indicating that *NCOA7* plays a role in suppressing the invasion and migration of ccRCC cells [14]. Additionally, experimental findings demonstrated that overexpressing *NCOA7* markedly diminishes the invasive and migratory capacities of ccRCC cells both in vitro and in vivo [14]. Patients with elevated *NCOA7* expression demonstrated a more favorable prognosis compared to those with low *NCOA7* expression in advanced stages of ccRCC [14]. Consistent with this study, we observed higher tumor grade and more advanced tumor stage according to the AJCC in ccRCC patients with *NCOA7* low expression.

Radiogenomic characteristics associated with *NCOA7* expression in ccRCC reflect the aggressive nature of the tumor. An ill-defined margin, tumor necrosis, and an exophytic growth pattern are well-established features linked to overall aggressiveness in ccRCC [19,20]. The occurrence of collateral vascular supply was strongly linked to high-grade tumors, more aggressive clinicopathological features, and poorer prognosis [21,22].

The identified radiogenomic features exhibit a specific pattern associated with *NCOA7* low expression in ccRCC.

The radiogenomic characteristics of *NCOA7* low expression share common features with Prolyl 4-Hydroxylase Subunit α 3 (*P4HA3*), specifically ill-defined margins and a more advanced tumor stage according to the AJCC [16]. However, *P4HA3* also exhibits traits such as larger primary tumor size and signs of infiltration, which are not observed in *NCOA7* low expression [16]. *NCOA7* shows primary tumor size at the threshold of statistical significance, while the remaining radiogenomic characteristics of *NCOA7* low expression are not observed in ccRCC with *P4HA3* expression [16].

Compared to ccRCC with adipose differentiation-related protein (*ADFP*) expression and the expression of GTPases of immunity-associated proteins (*GIMAP*) family genes, *NCOA7* low expression does not share any radiogenomic features. *ADFP* expression is associated with collecting system invasion, while the expression of *GIMAP* family genes is characterized by the absence of an endophytic growth pattern and the presence of infiltration [5,17].

The radiogenomic features of *ADAM12* expression in ccRCC include primary tumor size, ill-defined margins, tumor necrosis, and collecting system invasion [18]. *NCOA7* low expression shares ill-defined margins and tumor necrosis, while primary tumor size is at the threshold of statistical significance. However, it does not exhibit collecting system invasion.

BRCA1-associated protein 1 (ubiquitin carboxy-terminal hydrolase) (*BAP1*)-mutated ccRCC, in addition to ill-defined margins, also presents renal vein invasion and intratumoral calcifications [23,24]. While ccRCC with *BAP1* mutation shares ill-defined margins with ccRCC featuring *NCOA7* low expression, it differs by also exhibiting renal vein invasion and intratumoral calcifications.

In summary, *NCOA7* low expression in ccRCC was found to be significantly associated with several radiological and clinical features, including age, tumor grade, more advanced tumor stage according to the AJCC, collateral vascular supply, ill-defined margins, tumor composition, tumor necrosis, exophytic growth pattern ≥50%, and perinephric fat stranding. These results suggest that *NCOA7* low expression is associated with a radiologic phenotype indicative of increased tumor aggressiveness. The integration of genomic and radiological data using non-invasive methods provides valuable prognostic information, offering potential insights into gene expression pathways and paving the way for the development of targeted therapeutic strategies. This approach allows for the identification of high-risk patients and the personalization of therapeutic pathways, improving clinical management and optimizing the use of healthcare resources. Moreover, radiogenomic analysis enables monitoring of tumor evolution over time, supporting the timely adoption of more effective treatments. In this way, it is possible to achieve a more accurate characterization of tumor behavior, promoting clinical decisions based on integrated evidence from genetics and imaging.

This study has certain limitations that warrant consideration. First, the analysis relies on data from publicly available databases, which may introduce selection bias and limit the generalizability of the findings. The use of secondary data may lead to inconsistencies in imaging acquisition protocols. Additionally, the retrospective nature of the study restricts the ability to establish causality between *NCOA7* expression and the observed radiological and clinicopathological features. The sample size, although substantial, may still not fully capture the heterogeneity of ccRCC, particularly with respect to rare phenotypes. Moreover, the imaging features were evaluated using predefined criteria, which could introduce observer variability despite efforts to standardize assessments. The study also found a significant age difference between patients with high and low *NCOA7* expression, which could act as a confounding factor and may influence the observed associations. Finally, the lack of longitudinal data limits the ability to assess the dynamic changes in *NCOA7* expression and its correlation with treatment response or disease progression over time. Future prospective studies with larger, more diverse cohorts, better age-matched groups, and longitudinal follow-up are needed to validate and extend these findings.

## 5. Future Directions

The findings of this study offer significant insights into the radiogenomic characteristics associated with low *NCOA7* expression in ccRCC. However, given the retrospective nature and inherent limitations of the current research, it is necessary to explore several future directions to strengthen the understanding and clinical relevance of *NCOA7* in ccRCC.

First and foremost, future studies should aim to validate these findings through prospective, multicenter cohorts with larger and more diverse populations. Such studies would not only improve the generalizability of the results but also help mitigate biases introduced by the retrospective nature and use of secondary data. Including patients from diverse geographic locations and healthcare settings would allow for the identification of potential variations in *NCOA7* expression patterns and their associations with tumor aggressiveness and clinical outcomes.

Longitudinal studies are particularly needed to monitor changes in *NCOA7* expression over time and evaluate how these changes correlate with disease progression and therapy response. By incorporating follow-up data, researchers will gain a better understanding of the dynamic nature of *NCOA7* expression and its potential as a biomarker for monitoring treatment efficacy. This approach could help identify specific expression patterns that predict resistance to therapy or recurrence, allowing for early interventions and personalized therapeutic adjustments.

Moreover, functional studies are essential to investigate the molecular mechanisms underlying the role of *NCOA7* in tumor biology. While our study highlights the association between low *NCOA7* expression and aggressive radiological and clinical features, it remains crucial to elucidate the biological pathways through which *NCOA7* modulates tumor behavior. In vitro and in vivo experiments could examine how manipulating *NCOA7* expression influences cell proliferation, invasion, and metastasis. Additionally, investigating downstream signaling pathways, such as the mitogen-activated protein kinase/extracellular signal-regulated kinase pathway known to be modulated by *NCOA7*, could reveal targets for new therapeutic strategies [14].

Another important future direction involves further radiogenomic integration by combining imaging data with comprehensive multi-omics analyses, including transcriptomics, proteomics, and epigenomics [25,26,27,28,29]. This approach would offer a deeper understanding of how radiological features correlate with complex molecular alterations. An innovative aspect in this context is the use of radiomics, which allows for the automatic extraction of quantitative features from diagnostic images, providing an objective representation of tumor properties [30]. Combining radiomics with genomic data could reveal significant patterns that might be overlooked by traditional analysis methods.

Furthermore, the application of advanced computational techniques, such as machine learning and deep learning, could enhance the identification of complex radiogenomic patterns [31,32]. Machine learning models could be trained to recognize specific radiomic signatures associated with *NCOA7* expression levels, enabling the creation of robust predictive algorithms. Deep learning models, particularly convolutional neural networks, could be used to identify non-linear correlations between high-resolution radiological images and genetic profiles, improving diagnostic accuracy and clinical outcome prediction. These models could also support the automatic detection of the most relevant radiomic features, reducing the risk of errors due to subjective human interpretation.

The clinical application of *NCOA7* as a predictive biomarker requires the development of non-invasive diagnostic tools that leverage radiogenomic and radiomic data. Integrating *NCOA7*-related signatures into routine imaging protocols could facilitate risk stratification at diagnosis, guiding decision-making for targeted therapeutic interventions. To this end, it is crucial to establish standardized imaging criteria and develop automated algorithms capable of identifying the main radiogenomic and radiomic features associated with *NCOA7* expression.

Finally, exploring the potential of *NCOA7* as a therapeutic target remains a promising direction. Considering its role in suppressing tumor progression, therapies aimed at enhancing *NCOA7* expression or mimicking its function could provide new therapeutic options. Preclinical studies focusing on testing *NCOA7* agonists or gene therapy approaches could pave the way for innovative therapeutic strategies tailored to patients with low *NCOA7* expression.

In conclusion, future research should focus on validating current findings in larger cohorts, exploring the mechanistic basis of *NCOA7*’s role in tumor biology, and developing practical applications in diagnosis and treatment. By adopting a multidisciplinary and multi-omics approach that integrates radiomics and genomics, it will be possible to deepen the understanding of *NCOA7*’s impact on ccRCC and improve patient care through precision oncology.

## 6. Conclusions

*NCOA7* low expression in ccRCC is strongly associated with aggressive radiological and clinicopathological features, highlighting its potential as a biomarker for poor prognosis. Radiogenomic integration provides valuable insights into tumor behavior and may guide personalized treatment strategies. However, further prospective studies with larger, more diverse cohorts and longitudinal follow-up are needed to validate these findings and fully explore the clinical utility of *NCOA7*.

## Figures and Tables

**Table 1:** Descriptive characteristics of the study population stratified according to NCOA7 expression.

	Overall *n* = 209	NCOA7 Low Expression *n* = 138 (66.03%)	NCOA7 High Expression *n* = 71 (33.97%)	*p*-Value ^2^
Clinical–pathological features				
Age (years)	59 ^1^ (26–88)	61 ^1^ (26–85)	56 ^1^ (37–88)	**0.001**
Sex (males)	138 (66.03%)	96 (69.57%)	42 (59.15%)	0.177
Primary tumor size ^3^ (mm)	54 (20–165)	134 (20–144)	71 (15–165)	0.051
Laterality (right)	111 (53.11%)	71 (51.45%)	40 (56.34%)	0.60
Tumor grade (Fuhrman)				**0.015**
Low (G1-2)	84 (40.19%)	47 (34.06%)	37 (52.11%)	
High (G3-4)	138 (66.03%)	91 (65.94%)	34 (47.89%)	
Tumor stage ^3^ (AJCC)				**0.006**
Stage I	106 (51.21%)	61 (44.20%)	45 (64.29%)	
Stage II	20 (9.66%)	14 (10.14%)	6 (8.57%)	
Stage III	50 (24.15%)	35 (25.36%)	14 (20%)	
Stage IV	31 (14.98%)	28 (20.29%)	3 (4.29%)	
CT-based features				
Collateral vascular supply ^3^	112 (56.85%)	83 (63.36%)	29 (43.94%)	**0.014**
Tumor margins ^3^				**0.016**
Well defined	130 (64.36%)	78 (58.21%)	52 (76.47%)	
Ill defined	72 (35.64%)	56 (41.79%)	16 (23.53%)	
Tumor composition ^3^				**0.004**
Solid	187 (92.57%)	130 (97.01%)	57 (83.82%)	
Cystic	14 (6.54%)	4 (2.99%)	10 (14.71%)	
Tumor necrosis ^3^				**0.002**
0%	12 (5.91%)	3 (2.24%)	9 (13.24%)	
1–33%	120 (59.11%)	76 (56.72%)	44 (64.71%)	
34–66%	49 (24.63%)	37 (27.61%)	12 (17.65%)	
>66%	21 (10.34%)	18 (13.43%)	3 (4.41%)	
Tumor growth pattern ^3^				**0.002**
Endophytic	13 (6.37%)	6 (4.48%)	7 (10%)	
Exophytic <50%	60 (29.41%)	31 (23.13%)	30 (42.86%)	
Exophytic ≥50%	131 (64.22%)	97 (72.39%)	33(47.14%)	
Calcifications ^3^	39 (19.12%)	11 (5.39%)	28 (13.73%)	0.52
Signs of infiltrations ^3^	5 (2.45%)	4 (1.96%)	1 (0.49%)	0.83
Hydronephrosis ^3^	6 (2.98%)	2 (0.99%)	4 (1.99%)	0.23
Thrombosis or infiltration of renal artery ^3^	4 (2.02%)	3 (1.52%)	1 (0.51%)	1.0
Thrombosis or infiltration of renal vein ^3^	15 (7.39%)	11 (5.53%)	4 (2.01%)	0.692
Collecting system invasion ^3^	61 (30.65%)	44 (22.11%)	17 (8.54%)	0.238
Perinephric fat stranding ^3^	107 (53.5%)	79 (39.5%)	28 (14%)	**0.027**
Gerota’s fascia thickening ^3^	78 (39%)	57 (28.5%)	21 (10.5%)	0.155
Intralesional hemorrhage ^3^	4 (2.01%)	4 (2.01%)	0 (0%)	0.347

^1^ Median (IQR); ^2^ Student’s *t*-test; Pearson’s Chi-square test; ^3^ Data not available for all patients; Values in bold indicate statistical significance set at *p* < 0.05.

## Data Availability

The original contributions presented in the study are included in the article, further inquiries can be directed to the corresponding author.
